# Far‐Red/Near‐Infrared Conjugated Polymer Nanoparticles for Long‐Term In Situ Monitoring of Liver Tumor Growth

**DOI:** 10.1002/advs.201500008

**Published:** 2015-04-20

**Authors:** Jie Liu, Kai Li, Bin Liu

**Affiliations:** ^1^ Department of Chemical and Biomolecular Engineering4 Engineering Drive 4National University of Singapore117585Singapore; ^2^ Institute of Materials Research and Engineering3 Research Link117602Singapore

**Keywords:** conjugated polymer, far red/near‐infrared fluorescence, long‐term fluorescence imaging, monitoring of tumor growth, nanoparticle

## Abstract

The design and synthesis is reported for a fluorescent conjugated polymer (CP), poly{[4,4,9,9‐tetrakis(4‐(octyloxy)phenyl‐4,9‐dihydro‐*s*‐indaceno[1,2‐b:5,6‐b′]dithiophene)]‐*alt*‐*co*‐[4,7‐di(thiophen‐2‐yl)‐2,1,3‐benzothiadiazole]} (PIDT‐DBT), with absorption and emission profiles fallen within far‐red/near infrared (FR/NIR) region and further demonstrate its application in long‐term in vitro cell tracing and in vivo imaging of liver tumor growth. PIDT‐DBT‐Tat nanoparticles (NPs) have an absorption maximum at ≈600 nm with an emission maximum at ≈720 nm in water. In vitro cell tracing studies reveal that PIDT‐DBT‐Tat NPs can trace HepG2 liver cancer cells over 8 d. In vivo imaging results indicate that PIDT‐DBT‐Tat NPs can monitor liver tumor growth for more than 27 d in a real‐time manner. Both in vitro and in vivo studies demonstrate that PIDT‐DBT‐Tat NPs are superior to commercial Qtracker 705 as fluorescent probes. This study demonstrates for the first time the feasibility for long‐term in vivo imaging of tumor growth by utilizing CP‐based fluorescent probes, which will encourage the development of NIR fluorescent CPs for in vivo bioimaging.

## 1 Introduction

Cancer is one of the major causes of mortality in the world and the incidence of cancer continues to increase. Effective real‐time monitoring of tumor growth is critical to the success of cancer therapy and improvement of patient outcomes.^1^, ^2^, ^3^ However, evaluating tumor growth typically relies on the gross examination of tumor size through physical examination.^4^ This approach severely limits the use of clinically relevant orthotopic tumor models and gives little insight in the tumor processes being affected by the treatment. Therefore, development of techniques with ability to noninvasively image tumor growth processes would be beneficial in the evaluation of potential antitumor strategies. In vivo imaging technologies have emerged as indispensable tools in cancer research, clinical trials and medical practice, due to their abilities to visualize the location of tumors and assess their biological processes.^5^ Among in vivo studies, long‐term in situ imaging of tumors is of vital significance in precise diagnosis of cancer, image‐guided surgery, and monitoring of the treatment process.^6^ Despite the various available bioimaging techniques, such as magnetic resonance imaging,^7^ positron emission tomo­graphy,^8^ single photon emission computing tomo­graphy,^9^ radiography,^10^ and ultrasound,^11^ fluorescence imaging with easier maneuverability is an unique nonionizing technique in providing high sensitivity and spatiotemporal resolution.^12^, ^13^

To efficiently realize long‐term in situ in vivo fluorescence imaging of tumors, bright and stable near infrared (NIR) (650–900 nm) fluorescent probes are preferentially employed to accumulate within the site of interest through passive (such as enhanced permeability and retention effect) or active (binding to a receptor on the cell surface) targeting strategies.^14^, ^15^ Among them, organic fluorophores have been historically applied in in vivo applications due to their commercial availability and easy functionalization.^16^ However, their use for long‐term or real‐time in vivo imaging is hampered by their intrinsic drawbacks such as small Stokes shifts and poor photostability.^17^ As compared to discrete organic fluorophores, fluorescent nanoparticles (NPs) have advantages in prolonged intracellular retention,^18^ which is beneficial for long‐term studies. To date, several types of fluorescent NPs (noble metal NPs,^19^ upconversion NPs,^20^ and hybrid NPs^21^) have been employed for highly sensitive optical imaging of cancer at both cellular and animal levels. However, inorganic nanoparticles face the difficulties in biodegradability and some also show obvious toxicity.^22^, ^23^, ^24^ Fluorescent NPs with great biocompatibility and stable fluorescence in biological environments are preferential for long‐term in vivo imaging applications.

Fluorescent conjugated polymers (CPs) based NPs have recently received great attention in bioimaging applications.^25^, ^26^, ^27^ They have been used for targeted in vitro/in vivo cellular imaging, intracellular biomolecule imaging, and in vivo small molecule imaging due to their high brightness, good photostability, low cytotoxicity as well as readily tailored optical properties. Despite of their success in bioimaging, CP NPs have not been used for real‐time monitoring of tumor growth, largely due to the lack of highly emissive CPs with suitable absorption and emission profiles. The currently available CP based far‐red (FR)/NIR fluorescent NPs are mainly synthesized through chemical incorporation of narrow‐band‐gap moieties into CP backbones or physical blending of CP and FR/NIR fluorescent acceptors (e.g., organic dyes, quantum dots (QDs), and CPs).[[qv: 27c,d]],^28^ In these cases, the polymer absorption is strongly dependent on the donor component and the short wavelength is not suitable for in vivo bioimaging. Very recently, we have successfully shifted the absorption maxima of highly emissive FR/NIR fluorescent CP NPs to ≈488 and ≈530 nm, by simultaneous incorporation of two narrow‐band‐gap moieties into CP backbone to realize efficient intra‐ and intermolecular energy transfer.^29^

As part of our continuous efforts in developing bright FR/NIR CP NPs, in this contribution, we report the design and synthesis of poly{[4,4,9,9‐tetrakis(4‐(octyloxy)phenyl‐4,9‐dihydro‐*s*‐indaceno[1,2‐*b*:5,6‐*b*′]dithiophene)]‐*alt*‐*co*‐[4,7‐di(thiophen‐2‐yl)‐2,1,3‐benzothiadiazole]} (PIDT‐DBT). The polymer design takes into consideration that alternating strong electron‐rich (IDT) and electron‐deficient (DBT) units could lead to a strong intramolecular charge transfer band at long wavelength. In addition, the four 4‐(octyloxy)phenyl substitutions could minimize π stacking between conjugated backbones to favor high fluorescence in NPs. We further fabricated the CP NPs with cell penetrating peptide, human immunodeficiency virus type 1 (HIV‐1) trans‐activating transcriptional activator (Tat), on the surface (PIDT‐DBT‐Tat) and demonstrated their application in long‐term in vivo monitoring of liver tumor growth. In vitro cell tracing studies revealed that PIDT‐DBT‐Tat NPs can trace HepG2 liver cancer cells over 8 d. The NP‐labeled cells were further transplanted into the nude mice liver to monitor the liver tumor growth, which showed that the fluorescent signals can be clearly detected for 27 d. Both in vitro and in vivo studies reveal that PIDT‐DBT‐Tat NPs are superior to commercial Qtracker 705 as fluorescent probes. This study demonstrates for the first time the feasibility of using CP NPs for real‐time long‐term in vivo monitoring of tumor growth.

## 2 Results and Discussion

### 2.1 Synthesis and Characterization

**Scheme 1:**
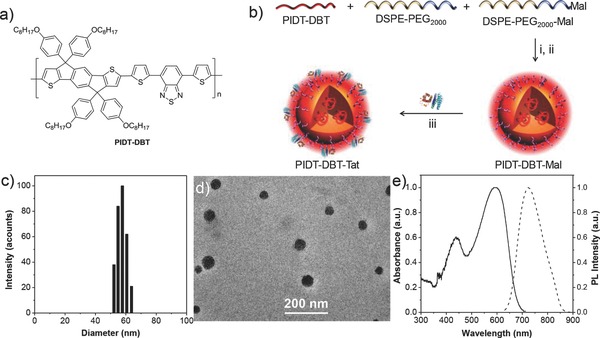
a) Chemical structure of PIDT‐DBT. b) Schematic illustration of PIDT‐DBT‐Tat NPs. Conditions: (i) water/THF, v:v = 10/1; (ii) sonication for 90 s, followed by solvent evaporation for 24 h; (iii) conjugation with HIV‐1 Tat peptide by click reaction. c) Particle size distribution of PIDT‐DBT‐Tat NPs studied by laser light scattering (LLS). d) TEM image of PIDT‐DBT‐Tat NPs. e) UV–vis absorption and PL spectra of PIDT‐DBT‐Tat NPs in water.

**Scheme** **1**a shows the chemical structure of PIDT‐DBT. The synthetic route is shown in Scheme S1, Supporting Information. In brief, 2,5‐dibromo‐*p*‐xylene was oxidized using KMnO_4_ as an oxidant to give dibromoterephthalic acid **1**, which was treated in absolute ethanol under acidic condition to form diethyl 2,5‐dibromoterephalate **2**. Stille coupling reaction between **2** and 2‐tributylstannyl thiophene was performed to afford diethyl 2,5‐di(thien‐2‐yl)terephthalate **3**, which was first reacted with 4‐octyloxyphenyllithum salt at −78 °C, and then treated under acidic condition to afford 4,4,9,9‐tetrakis(4‐(octyloxy)phenyl)‐4,9‐dihydro‐*s*‐indaceno[1,2‐*b*:5,6‐*b*′]dithiophene **4**. 2,7‐Dibromo‐4,4,9,9‐tetrakis(4‐(octyloxy)phenyl)‐4,9‐dihydro‐*s*‐indaceno[1,2‐*b*:5,6‐*b*′]dithiophene **5** was obtained through bromination of **4**. 4,7‐Bis(5‐(trimethylstannyl)thiophen‐2‐yl)‐2,1,3‐benzothiadiazole **6** was synthesized according to previous report.^30^ Stille polymerization between **5** and **6** was carried out to give PIDT‐DBT with careful purification. The chemical structures of all the intermediates and PIDT‐DBT were verified by nuclear magnetic resonance (NMR) spectra. The number average molecular weight and polydispersity index of PIDT‐DBT are 18 700 and 1.50, respectively, determined by gel permeation chromatography (GPC) using tetrahydrofuran (THF) as eluent and polystyrene as standard. PIDT‐DBT has good solubility in common organic solvents, e.g., toluene, dichloromethane, and tetrahydrofuran.

### 2.2 Fabrication and Characterization of NPs

Scheme 1b shows the schematic illustration of the fabrication of PIDT‐DBT‐Tat NPs. The NPs were fabricated by a modified nanoprecipitation method.^31^ More specifically, 1,2‐Distearoyl‐*sn*‐glycero‐3‐phosphoethanolamine‐*N*‐[methoxy(polyethylene glycol)‐2000] (DSPE‐PEG_2000_) and its maleimide modified derivative DSPE‐PEG_2000_‐Mal were chosen as matrix. Upon addition of a well dissolved DSPE‐PEG_2000_/DSPE‐PEG_2000_‐Mal/PIDT‐DBT (1/1/1) mixture in THF solution into tenfold Milli‐Q water under continuous ultrasonication, the hydrophobic PIDT‐DBT and DSPE components are likely to be embedded into the particle core while the hydrophilic PEG and PEG‐Mal segments are extended outside into the aqueous environments to render the NPs with abundant maleimide groups on the surface. The PIDT‐DBT‐Mal NPs were then conjugated with cell penetrating peptide HIV‐1 Tat (RKKRRQRRRC) to afford PIDT‐DBT‐Tat NPs via click coupling reaction between the maleimide groups on NP surface and thiol groups of peptides at the C‐turminus. The obtained nanoparticles were kept in refrigerator at 4 °C, and no obvious suspensions were observed for 2 months.

The volume average hydrodynamic diameters of PIDT‐DBT‐Tat NPs were studied by laser light scattering (LLS), revealing that PIDT‐DBT‐Tat NPs have a volume average hydrodynamic diameter of ≈56 nm with a narrow size distribution (Scheme 1c). The size and morphology of PIDT‐DBT‐Tat NPs were further studied by transmission electron microscopy (TEM). As shown in Scheme 1d, the NPs are clearly distinguished with a spherical shape, which have a mean diameter of ≈49 nm. The slightly smaller size relative to that revealed by LLS is due to shrinkage of polymeric NPs in dry state.^32^ In addition, the hydrodynamic diameter of PIDT‐DBT‐Tat NPs does not show any obvious change even after incubation at 37 °C in phosphate‐buffered saline (PBS, pH = 7.4) solution for 14 d, suggesting that PIDT‐DBT‐Tat NPs have excellent colloidal stability (Figure S1, Supporting Information). Scheme 1e shows the UV–vis absorption and photoluminescence (PL) spectra of PIDT‐DBT‐Tat NPs in water. The NPs have an absorption peak at ≈600 nm with an emission maximum at ≈720 nm, which gives rise to a remarkably large Stoke shift of ≈120 nm, endowing the NPs with great potentials to minimize background interference for in vivo bioimaging application. The absorption spectrum also has a long tail extending over 750 nm, allowing excitation in the FR/NIR region, which is a great advantage for in vivo imaging.

### 2.3 In Vitro Cell Tracing

**Figure 1:**
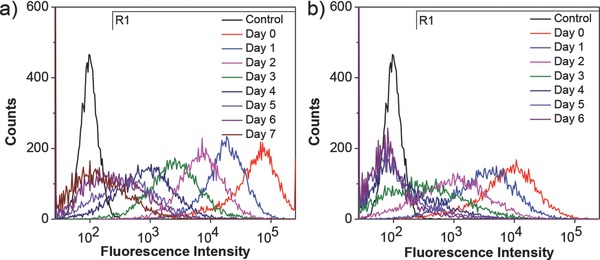
Flow cytometry histograms of HepG2 cancer cells after incubation with 2 × 10^−9^ m: a) PIDT‐DBT‐Tat NPs and b) Qtracker 705 at 37 °C for 24 h and then subcultured for designated days. The untreated HepG2 cells were used as the control.

To evaluate the performance of PIDT‐DBT‐Tat NPs as a fluorescent probe, we first studied their in vitro cell tracing ability. HepG2 liver cancer cells were chosen as a model cell line and Qtracker 705 Labeling Kit was used as the benchmark due to its matched emission maximum with PIDT‐DBT‐Tat NPs. For in vitro cell tracing studies, the HepG2 cancer cells were first incubated with 2 × 10^−9^ m PIDT‐DBT‐Tat NPs and Qtracker 705 at 37 °C for 24 h. The labeled cells were then subcultured in the absence of the probe for designated days and the fluorescence profiles were studied using flow cytometry by counting 10 000 events (*λ*_ex_ = 561 nm, 710/50 nm bandpass filter). As shown in **Figure** **1**a, the efficient labeling rate of HepG2 cells upon incubation with PIDT‐DBT‐Tat NPs remains ≈100% till day 2 as compared to the untreated cells. The labeling rate drops to 97.3% and 78.1% after continuous culture for 3 and 4 d, respectively. After 5 d, 53.1% of the cells are still efficiently labeled, while the labeling rate is 23.5% at day 7. On the contrary, only 75.9% and 43.4% of Qtracker 705‐treated cells are effectively labeled at day 2 and day 3, while the labeling rate further drops to 18.1% and 4.6% at day 4 and day 6 (Figure 1b), respectively. Compared to Qtracker 705, these results suggest that PIDT‐DBT‐Tat NPs are more suitable for long‐term cell tracing studies with higher fluorescence intensity and longer tracing period.

**Figure 2:**
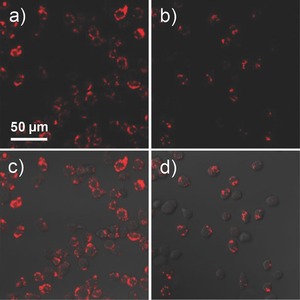
CLSM fluorescence images of HepG2 cancer cells after incubation with 2 × 10^−9^ m: a) PIDT‐DBT‐Tat NPs and b) Qtracker 705 at 37 °C for 24 h. c,d) The corresponding fluorescence/transmission overlay images. All images share the same scale bar (*λ*_ex_ = 561 nm, 710/50 nm bandpass filter).

The efficient labeling of HepG2 cells after incubation with PIDT‐DBT‐Tat NPs was further confirmed by confocal laser scanning microscopy (CLSM). As shown in **Figure** **2**a, nearly all the cells in suspension show intense fluorescence after incubation with 2 × 10^−9^ m PIDT‐DBT‐Tat NPs for 24 h (day 0 sample for flow cytometry studies). On the contrary, upon incubation with 2 × 10^−9^ m Qtracker 705 (Figure 2b), some cells show very dim fluorescence signal and the average intensity is weaker as compared to that incubated with PIDT‐DBT‐Tat NPs. The 3D color‐coded projection of PIDT‐DBT‐Tat NP‐labeled cells was also obtained, revealing that the NPs are mainly distributed in cytoplasm (Figure S2, Supporting Information). In addition, we also studied the confocal imaging of cells incubated with 2 × 10^−9^ m of PIDT‐DBT NPs (without Tat) under the same experimental conditions. As shown in Figure S3, Supporting Information, only very weak fluorescence intensity can be detected from the PIDT‐DBT NP‐treated cells (Figure S3b, Supporting Information) while much stronger fluorescence signal from the PIDT‐DBT‐Tat NP‐treated cells was observed, suggesting the much lower internalization efficiency of the nonfunctionalized NPs. The results indicate that the conjugation of Tat peptide on NP surface is essential to enhance the internalization of NPs into living cells.^33^ Moreover, the cytotoxicity of PIDT‐DBT‐Tat NPs was evaluated using methylthiazolyldiphenyl‐tetrazolium bromide (MTT) assay. As shown in Figure S4, Supporting Information, the metabolic viabilities of HepG2 and NIH/3T3 cells show negligible difference and remain above 90% after incubation with 2, 4, and 8 × 10^−9^ m PIDT‐DBT‐Tat NPs for 48 h. The viability of NIH/3T3 cells after incubation with 72 h still remains above 85% (Figure S5, Supporting Information), suggesting that the NPs are of low cytotoxicity during the test period, which is beneficial to long‐term in vivo studies.

### 2.4 In Vivo Monitoring of Liver Tumor Growth

**Figure 3:**
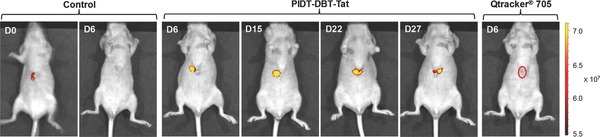
Representative in vivo fluorescence images of the mouse transplanted with 4 × 10^6^ of HepG2 cells labeled by PIDT‐DBT‐Tat NPs and Qtracker 705. The control images were obtained from a nude mouse that underwent the same surgical operation without injection of labeled HepG2 cells. The images were taken on designated days postcell injection (*λ*_ex_ = 640 nm, 720/20 nm filter).

The ability of PIDT‐DBT‐Tat NPs for real‐time in vivo monitoring of liver tumor growth was subsequently evaluated. Surgical operation was conducted at the abdomen of Balb/c nude mice and 4 × 10^6^ of HepG2 cells labeled with 2 × 10^−9^ m PIDT‐DBT‐Tat NPs or Qtracker 705 were directly injected into the parenchymal cells of the left lobe of liver (two groups, *n* = 3 for each group). Meanwhile, a nude mouse that underwent the same surgical operation without injection of labeled HepG2 cells was used as a control. After injection and suture, the in vivo fluorescence images of these mice were recorded using an IVIS Spectrum Imaging System. As shown in **Figure** **3**, the suture at surgical site shows intense fluorescence upon excitation at 640 nm. After 6 d, the suture was totally absorbed and the cut closed up with no background fluorescence detectable from the surgical site. As shown in Figure 3, the mice injected with PIDT‐DBT‐Tat NP‐labeled HepG2 cells shows intense fluorescence at the surgical site at day 6, due to the high labeling efficiency and efficient NIR emission of PIDT‐DBT‐Tat NPs. The fluorescence signal remains detectable even after 27 d. On the contrary, no fluorescence can be detected from the mice injected with Qtracker 705‐labeled cells at day 6 under the same experimental setup. These results suggest that PIDT‐DBT‐Tat NPs can serve as a long‐term in vivo tumor tracing probe with advantages over commercial Qtracker 705 in real‐time fluorescence imaging.

**Figure 4:**
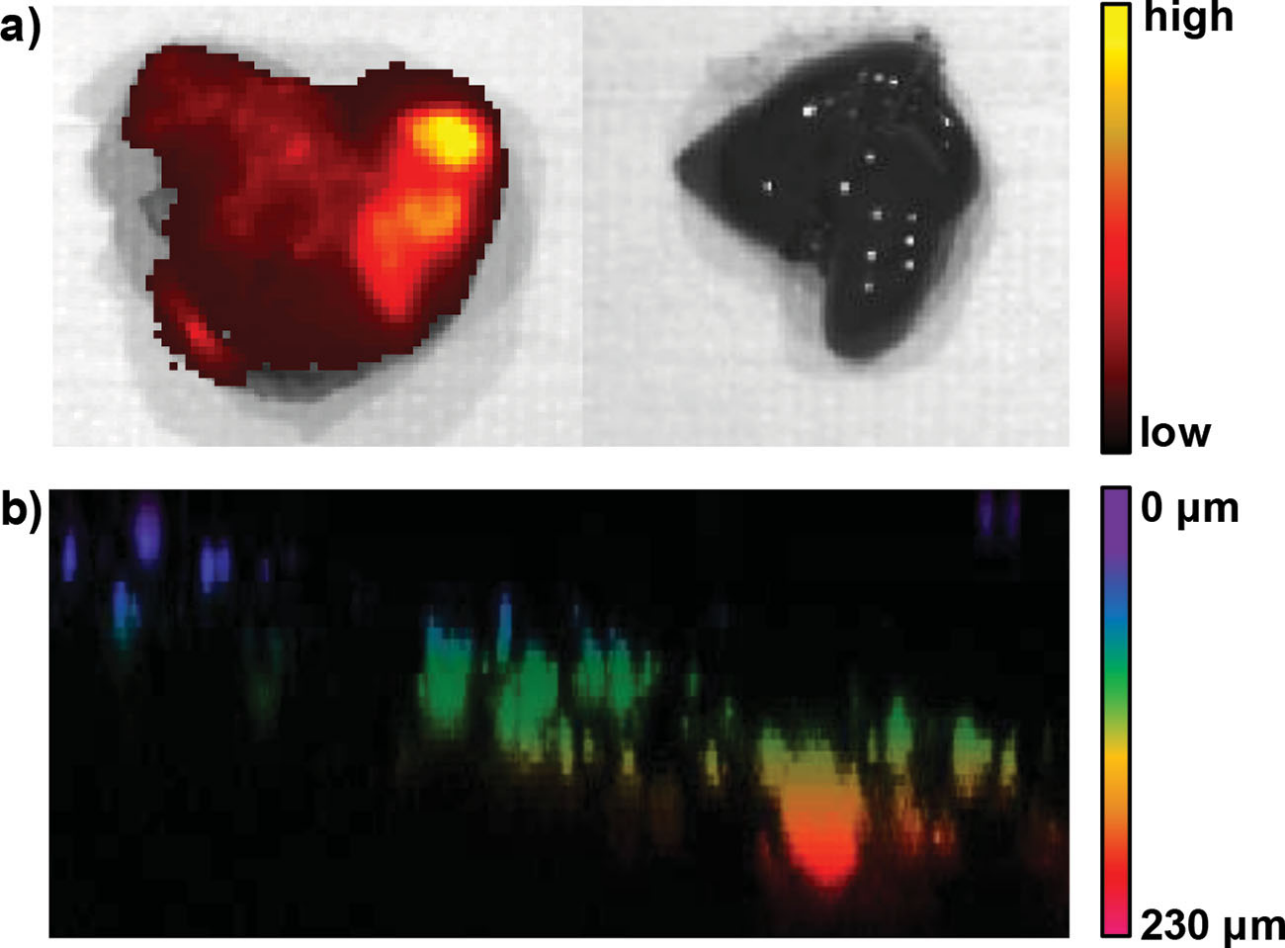
a) Representative ex vivo fluorescence images of the liver collected from mouse transplanted with 4 × 10^6^ of PIDT‐DBT‐Tat NP‐labeled HepG2 cells (left) and mouse without transplantation (right) after 42‐day injection (*λ*_ex_ = 640 nm, 720/20 nm filter). b) 3D color‐coded projection of z‐stacks of confocal images (*λ*_ex_ = 590 nm, 600–800 nm bandpass filter).

Upon 42 d postinjection of PIDT‐DBT‐Tat NP‐labeled HepG2 cells, one mouse was sacrificed to collect the liver tissue. It can be clearly seen that the transplanted cancer cells have grown into solid tumors (Figure S6, Supporting Information) and spread all over the whole liver to emit intense fluorescent signal (**Figure** **4**a). On the contrary, the liver tissues from mouse without HepG2 cell transplantation show no fluorescent signal under the same experimental conditions. These results indicate the ability of PIDT‐DBT‐Tat NPs for tracing tumor growth during cancer cell proliferation and invasion without detectable interference in the process. The whole tumor was then mounted and imaged upon excitation at 590 nm using one‐photon excited fluorescence microscope. The efficient penetration depth of fluorescence from PIDT‐DBT‐Tat NPs in tumor tissues was studied by taking images layer‐by‐layer at a 3 μm interval. The 3D color‐coded projection of deep tissue image reveals that the fluorescent signal from the NP‐labeled cells can be detected at 230 μm depth in the liver tumor upon excitation at 590 nm, proving the ability of the NIR emissive PIDT‐DBT‐Tat NPs in deep tissue imaging.

The ex vivo fluorescence imaging of organs (liver, heart, intestine, spleen, kidney, and lung) at day 1 and day 7 after cancer cell transplantation was also performed as shown in Figure S7, Supporting Information. It shows that no fluorescence could be detected from other organs except liver, suggesting the majority of PIDT‐DBT‐Tat NPs labeled cancer cells remain in the liver and negligible amount of them migrate to other organs during the test period. As the cells are directly injected to a particular site on liver (parenchymal cells) during the experiments, they prefer homing in the organ but not migrating to other organs.

## 3 Conclusion

To conclude, we report the design and synthesis of a fluorescent conjugated polymer PIDT‐DBT with absorption and emission profiles fallen within FR/NIR region. We further demonstrated the preparation of their NIR emissive NPs for long‐term in vitro cell tracing and in vivo imaging of liver tumor growth. PIDT‐DBT‐Tat NPs show an absorption maximum at ≈600 nm and an emission maximum at ≈720 nm in water, which is the first CP used for in vivo cancer cell tracking. Our studies have shown that PIDT‐DBT‐Tat NPs are superior to Qtracker 705 as fluorescent probes. In vivo imaging results indicate that PIDT‐DBT‐Tat NPs can monitor liver tumor growth for more than 27 d in a real‐time manner. This study demonstrates for the first time the feasibility of utilizing CP‐based fluorescent probes for long‐term in vivo imaging of tumor growth, which will encourage the development of NIR fluorescent CPs for in vivo bioimaging.

## 4 Experimental Section

*Materials*: 1,4‐Dibromo‐2,5‐dimethylbenzene, potassium permanganate (KMnO_4_), *tert*‐butyl alcohol, 2‐(tributylstannyl)thiophene, bis(triphenylphosphine)palladium(II) dichloride (Pd(PPh_3_)_2_Cl_2_), tetrakis(triphenylphosphine)palladium(0) (Pd(PPh_3_)_4_), *n*‐butyl lithium (*n*‐BuLi), *N*‐bromosuccinimide (NBS), 3‐(4,5‐dimethylthiazol‐2‐yl)‐2,5‐diphenyltetrazolium bromide (MTT) were purchased from Sigma‐Aldrich and used as received. Cell penetrating peptide HIV‐1 Tat was purchased from Genicbio, China. Dulbecco's modified eagle's medium (DMEM) was a commercial product of National University Medical Institutes (Singapore). Toluene used for polymerization was pretreated with sulfuric acid followed by distillation. All other chemicals were used as received, unless otherwise stated.

*Characterization*: Nuclear magnetic resonance (NMR) spectra were recorded on a Bruker Avance 500 NMR spectrometer (500 MHz for ^1^H, referenced to tetramethylsilane (TMS) at *δ* = 0.00 ppm and 125 MHz for ^13^C, referenced to CDCl_3_ at 77.0 ppm). Gel permeation chromatography (GPC) analysis was carried out with Waters 996 photodiode detector and Phenogel GPC columns, using polystyrenes as the standard and THF as the eluent at a flow rate of 1.0 mL min^−1^ at 35 °C. Laser light scattering (LLS) measurements were performed using Brookhaven instruments corporation (BIC) 90 plus with λ = 659 nm, and the particle diameters were calculated by ZetaPlus Particle Sizing Software Version 3.93. Transmission electron microscopy (TEM) studies were performed on a JEOL JEM‐2010 electron microscope with an accelerating voltage of 200 kV. UV–vis spectra were collected on a Shimadzu UV‐1700 spectrometer. Photoluminescence (PL) spectra were measured on a Perkin Elmer LS‐55 equipped with a xenon lamp excitation source and a Hamamatsu (Japan) 928 PMT, using 90° angle detection for solution samples. All UV–vis and PL spectra were collected at 24 ± 1 °C. Fluorescence quantum yield was measured using rhodamine 6G in methanol (95%) as the standard. The absorbance of the solutions was kept below 0.1 to avoid internal filter effect. Confocal laser‐scanning microscopy (CLSM) images were collected on a Zeiss LSM 410 (jena, Germany) CLSM with imaging soft (Fluoview FV1000). MilliQ water (18.2 MQ) was used for all the experiments.

*2,5‐Dibromoterephthalic Acid (**1**)*: To a suspension of 2,5‐dibromo‐*p*‐xylene (10.0 g, 37.8 × 10^−3^ m) and celite (15 g) in a mixture of water/*tert*‐BuOH (100/100 mL) was added KMnO_4_ (29.9 g, 189.4 × 10^−3^ m) in portions within 30 min. The mixture was stirred at 100 °C for 36 h. After cooling to 70 °C, ethanol (40 mL) was added slowly to remove unreacted KMnO_4_. The reaction mixture was filtered off and the filtrate was concentrated to ≈20 mL under reduced pressure. The residue was acidified by addition of concentrated HCl (10 mL). The precipitate was collected by filtration and washed with cold ethanol to give **1** as a white crystal (11.2 g, yield: 91%). The product was directly used in the next step without further purification.

*Diethyl 2,5‐Dibromoterephalate (**2**)*: To a solution of 2,5‐dibromoterephthalic acid (10.0 g, 30.8 × 10^−3^ m) in absolute ethanol (50 mL) was added concentrated H_2_SO_4_ (15 mL). The mixture was refluxed overnight before being cooled to room temperature. The precipitate was collected by filtration and further purified by recrystallization from ethanol to give **2** as a white crystal (10.9 g, yield: 93%). ^1^H NMR (600 MHz, CDCl_3_, ppm) δ: 8.02 (s, 2 H), 4.41 (q, *J* = 7.2 Hz, 4 H), 1.41 (t, *J* = 7.2 Hz, 6 H). ^13^C NMR (150 MHz, CDCl_3_, ppm) δ: 160.20, 136.40, 135.69, 120.05, 62.33, 14.13.

*Diethyl 2,5‐Di(thien‐2‐yl)terephthalate (**3**)*: To a solution of diethyl 2,5‐dibromoterephthalate (4.3 g, 11.3 × 10^−3^ m) and Pd(PPh_3_)_2_Cl_2_ (397.1 mg, 565.7 × 10^−6^ m) in anhydrous THF (15 mL) was added 2‐(tributylstannyl)thiophene (10.8 mL, 33.9 × 10^−3^ m). The reaction mixture was stirred at 85 °C under argon atmosphere for 15 h before being cooled to room temperature. The mixture was treated with potassium fluoride aqueous solution (10 wt%, 50 mL) for 2 h. The product was extracted with dichloromethane three times and the combined organic phase was washed with water three times and then dried over MgSO_4_. After filtration, the solvent was removed under reduced pressure. The residue was purified by silica gel chromatography (hexane/ethyl acetate = 9/1) to afford **3** as a white solid (3.9 g, yield: 90%). ^1^H NMR (600 MHz, CDCl_3_, ppm) δ: 7.81 (s, 2 H), 7.39 (dd, *J* = 4.8, 1.2 Hz, 2 H), 7.08 (m, 4 H), 4.21 (q, *J* = 7.2 Hz, 4 H), 1.15 (t, *J* = 7.2 Hz, 6 H). ^13^C NMR (150 MHz, CDCl_3_, ppm) δ: 167.65, 140.46, 134.05, 133.41, 131.84, 127.30, 126.94, 126.43, 61.62, 13.78.

*4,4,9,9‐Tetrakis(4‐(octyloxy)phenyl)‐4,9‐dihydro‐s‐indaceno[1,2‐b:5,6‐b*′*]dithiophene (**4**)*: A solution of 1‐bromo‐4‐octyloxybenzene (9.975 g, 35.0 × 10^−3^ m) in anhydrous THF (70 mL) was treated with *n*‐BuLi (21.87 mL, 1.6 m in hexane, 35 × 10^−3^ m) at –78 °C for 1 h. Then a solution of diethyl 2,5‐di(thien‐2‐yl)terephthalate (1.93 g, 5.0 × 10^−3^ m) in anhydrous THF (10 mL) was added slowly into the reaction mixture. After the addition was completed, the reaction mixture was stirred at –78 °C for 1 h and then kept at room temperature for 12 h. The solution was quenched with water (20 mL). After solvent removal, the residue was subsequently extracted with dichloromethane (100 mL × 3), washed with water (100 mL × 3) and dried over MgSO_4_. The organic layer was collected and concentrated to remove the solvents. The residue was dissolved in boiling acetic acid (100 mL) followed by the addition of concentrated HCl (10 mL). The mixture was allowed to reflux for 3 h and then quenched with water (100 mL). After extraction with dichloromethane, the combined organic phase was concentrated and purified by silica gel chromatography (hexane/ethyl acetate = 9/1) to afford **4** as a white solid (4.2 g, yield: 78%). ^1^H NMR (600 MHz, CDCl_3_, ppm) δ: 7.39 (s, 2 H), 7.24 (d, *J* = 4.8 Hz, 2 H), 7.15 (d, *J* = 9 Hz, 8 H), 6.97 (d, *J* = 4.8 Hz, 2 H), 3.90 (t, *J* = 6 Hz, 8 H), 1.75 (m, 8 H), 1.41 (m, 8 H), 1.28 (m, 32 H), 0.88 (t, *J* = 7.2 Hz, 12 H). ^13^C NMR (125 MHz, CDCl_3_, ppm) δ: 157.95, 156.14, 153.73, 141.00, 136.69, A34.97, 128.98, 127.46, 122.88, 117.23, 114.13, 67.89, 61.88, 31.79, 29.33, 29.28, 29.21, 26.05, 22.63, 14.07

*2,7‐Dibromo‐4,4,9,9‐tetrakis(4‐(octyloxy)phenyl)‐4,9‐dihydro‐s‐indaceno[1,2‐b:5,6‐b*′*]dithiophene (**5**)*: To a solution of 4,4,9,9‐tetrakis(4‐(octyloxy)phenyl)‐4,9‐dihydro‐*s*‐indaceno[1,2‐*b*:5,6‐*b*′]dithiophene (3.50 g, 3.23 × 10^−3^ m) in THF/*N*,*N*‐dimethylformamide (DMF) (2/1, 75 mL) was added NBS (1.26 g, 7.11 × 10^−3^ m) in portions within 20 min. This mixture was stirred for 3 h in the presence of light at room temperature and then the solvent was removed under reduced pressure. The residue was subsequently dissolved in dichloromethane (200 mL), washed with water (3 × 100 mL) and dried over MgSO_4_. After solvent removal, the residue was purified by silica gel chromatography (hexane/dichloromethane = 7/3) to afford **5** as a white solid (3.80 g, yield: 94%). ^1^H NMR (600 MHz, CDCl_3_, ppm) δ: 7.31 (s, 2 H), 7.14 (d, *J* = 8.5 Hz, 8 H), 6.98 (s, 2 H), 6.80 (d, *J* = 8.5 Hz, 8 H), 3.92 (t, *J* = 6 Hz, 8 H), 1.76 (m, 8 H), 1.44 (m, 8 H), 1.32 (m, 32 H), 0.90 (t, *J* = 7.2 Hz, 12 H). ^13^C NMR (150 MHz, CDCl_3_, ppm) δ: 158.65, 155.61, 153.35, 141.57, 136.30, 135.33, 129.32, 126.38, 117.47, 114.78, 114.19, 68.41, 63.18, 32.21, 29.74, 29.63, 26.48, 23.04, 14.47.

*Poly{[4,4,9,9‐tetrakis(4‐(octyloxy)phenyl‐4,9‐dihydro‐s‐indaceno[1,2‐b:5,6‐b*′*]dithiophene)]‐alt‐co‐[4,7‐di(thiophen‐2‐yl)‐2,1,3‐benzothiadiazole]}(PIDT‐DBT)*: A Schlenk tube was charged with 2,7‐dibromo‐4,4,9,9‐tetrakis(4‐(octyloxy)phenyl)‐4,9‐dihydro‐s‐indaceno[1,2‐b:5,6‐b′]dithiophene (124.1 mg, 0.1 × 10^−3^ m), 4,7‐bis(5‐(trimethylstannyl)thiophen‐2‐yl)‐2,1,3‐benzothiadiazole (62.6 mg, 0.1 × 10^−3^ m) and Pd(PPh_3_)_4_ (5 mg, 4.0 × 10^−6^ m) before it was sealed with a rubber septum. The Schlenk tube was degassed with three freeze‐pump‐thaw cycles to remove air. Then, toluene (8 mL) was added to the Schlenk tube and the mixture was frozen, evacuated, and thawed three times to further remove air. After the mixture was kept at 100 °C for 24 h, the reaction was stopped and cooled down to room temperature. The mixture was dropped slowly into methanol (100 mL) to precipitate the crude polymer followed by centrifugation. The crude polymer was subsequently redissolved in dichloromethane (200 mL), washed with water three times, and dried over MgSO_4_. After solvent removal, PIDT‐DBT (97.9 mg, yield: 71%) was obtained as a black solid by precipitation in methanol. ^1^H NMR (500 MHz, CDCl_3_, ppm) δ: 7.95 (br), 7.76 (br), 7.31 (br), 7.14 (br), 7.12 (br), 6.72 (br), 3.86 (br), 1.69 (br), 1.43 (br), 1.35 (br), 1.22 (m, br), 0.80 (br).

*Synthesis of PIDT‐DBT‐Tat NPs*: A THF solution (1 mL) containing PIDT‐DBT (1 mg) and a mixture of DSPE‐PEG_2000_ (1 mg) and DSPE‐PEG_2000_‐Mal (1 mg) was mixed with water (10 mL), followed by sonication for 60 s using a microtip probe sonicator at 12 W output (XL2000, Misonix Incorporated, NY). The mixture was stirred vigorously overnight in fumehood to evaporate THF. The CP NPs were further reacted with HIV1‐Tat peptide (1 × 10^−6^ m) via click coupling reaction overnight. The solution was then dialyzed against MilliQ water for 2 d to eliminate the excess Tat peptide. After filtration using a 0.2 μm syringe driven filter, the PIDT‐DBT‐Tat NPs were collected for further use.

*Cell Culture*: HepG2 human liver cancer cells were cultured in DMEM containing 10% fetal bovine serum and 1% penicillin streptomycin at 37 °C in a humidified environment containing 5% CO_2_. Before experiment, the cells were precultured until confluence was reached.

*Cytotoxicity of PIDT‐DBT‐Tat NPs*: The metabolic activities of HepG2 cells were evaluated using methylthiazolyldiphenyltetrazolium bromide (MTT) assays. Cells were seeded in 96‐well plates (Costar, IL, USA) at an intensity of 4 × 10^4^ cells mL^−1^, respectively. After 24 h incubation, the old medium was replaced by PIDT‐DBT‐Tat NPs suspension at concentrations of 2, 4, and 8 × 10^−9^ m, and the cells were then incubated for 48 h, respectively. To eliminate the UV–vis absorption interference of the PIDT‐DBT‐Tat NPs at 570 nm, the cells incubated with the PIDT‐DBT‐Tat NPs without posttreatment by MTT were used as the control. After designated time intervals, the wells were washed twice with 1× PBS buffer and 100 μL of freshly prepared MTT (0.5 mg mL^−1^) solution in culture medium was added into each well. The MTT medium solution was carefully removed after 3 h incubation in the incubator. Dimethyl sulfoxide (DMSO) (100 μL) was then added into each well and the plate was gently shaken for 10 min at 24°C to dissolve all the precipitates formed. The absorbance of MTT at 570 nm was monitored by the microplate reader (Genios Tecan). Cell viability was expressed by the ratio of the absorbance of the cells incubated with PIDT‐DBT‐Tat NP suspension to that of the cells incubated with culture medium only. The viability of NIH/3T3 cells after incubation with PIDT‐DBT‐Tat NPs for 48 and 72 h were also carried out following the same procedures.

*In Vitro Cell Tracing*: HepG2 human liver cancer cells were cultured in 6‐well plates (Costar, IL, USA) to achieve 80% confluence. After medium removal and washing with 1× PBS buffer, 2 × 10^−9^ m PIDT‐DBT‐Tat NPs or Qtracker 705 in DMEM medium were then added to the wells. After incubation at 37 °C for 24 h, the cells were washed with 1× PBS buffer and detached by 1× tripsin and resuspended in culture medium. Upon dilution, the cells were subcultured in 6‐well plates containing cell culture coverslips for 2, 4, and 6 d, respectively. After designated time intervals, the cells were trypsinalized to be suspend and fixed with 4% paraformaldehyde for 15 min. The fluorescence intensities of cells were analyzed by flow cytometry measurements using Cyan‐LX (DakoCytomation) and the histogram of each sample was obtained by counting 10 000 events (*λ*_ex_ = 561 nm, 710/50 nm bandpass filter). A batch of blank cells without any treatment was used as the control group. The suspended cells from the day 0 sample for flow cytometry test were also imaged using Leica TCS SP 5X upon excitation at 590 nm with a 600–800 nm bandpass filter.

*Animals*: Male Balb/c nude mice were obtained from the Biological Resource Centre (Biopolis, Singapore). Mice were housed in groups (5 per cage) and provided with standard mouse chow and water at libitum. The cages were maintained in a room with controlled temperature (25 ± 1 °C) and a 12 h light/dark cycle (light on at 7:00 am). All animal experiments were performed in compliance with guidelines set by the Institutional Animal Care and Use Committee (IACUC), SingHealth.

*In Vivo Cell Tracing*: HepG2 cells were incubated with 2 × 10^−9^ m PIDT‐DBT‐Tat NPs or Qtracker 705 overnight at 37 °C. The cells were then trypsinalized and resuspended in DMEM medium at 2 × 10^7^ cells mL^−1^. Mice were anaesthetized with intraperitoneal injection of 50/5 mg kg^−1^ of ketamine/diazepam solution followed by intramuscular injection of 5 mg kg^−1^ of baytril preoperatively. The surgical site at abdomen was cleaned and a midline incision was made in the abdomen to expose the left lobe of liver. The cell suspension was directly injected into the parenchymal cells of the liver. Abdomen was then sutured with 5/0 maxon (monofilament polyglyconate synthetic absorbable suture) and skin closed with 5/0 prolene (polypropylene suture). Mice were kept warm after the surgery and returned to their cages when fully awake. After designated time intervals postsurgical operation, the mice were imaged using an IVIS Spectrum Imaging System (Xenogen Co., Alameda, CA, USA) while under anesthesia using 1%–2% of isoflurane in oxygen. The fluorescence images were recorded with 1 s exposure using a filter 720/20 nm upon excitation at 640 nm. The autofluorescence was removed using the software of IVIS Spectrum Imaging System.

*Ex Vivo One‐Photon Excited Fluorescence Imaging*: In the ex vivo tumor imaging experiment, the mice were euthanized with CO_2_ and the livers were collected at 42 d postinjection and fixed in 4% paraformaldehyde for one‐photon excited 3D fluorescence imaging. The tumor was resected and fixed in 4% paraformaldehyde for 2 h and imaged using Leica TCS SP 5X. Confocal images of consecutive layers with approximately 3 μm interval were recorded to generate 3D colored projection to demonstrate the penetration depth of PIDT‐DBT‐Tat NPs in the tumor. The confocal images were taken upon excitation at 590 nm with a 600–800 nm bandpass filter.

*Biodistribution Studies*: The mice injected with PIDT‐DBT‐Tat NP‐labeled HepG2 cells were euthanized with CO_2_ and the organs (liver, heart, intestine, spleen, kidneys, and lung) were harvested at 1 d and 7 d postsurgery, respectively. The collected organs were then imaged using an IVIS Spectrum Imaging System (Xenogen Co., Alameda, CA, USA). The fluorescence images were recorded with 1 s exposure using a filter 720/20 nm upon excitation at 640 nm. The autofluorescence was removed using the software of IVIS Spectrum Imaging System.

## Supporting information

As a service to our authors and readers, this journal provides supporting information supplied by the authors. Such materials are peer reviewed and may be re‐organized for online delivery, but are not copy‐edited or typeset. Technical support issues arising from supporting information (other than missing files) should be addressed to the authors.

Supplementary
