# Supplementary material for: Far‐Red/Near‐Infrared Conjugated Polymer Nanoparticles for Long‐Term In Situ Monitoring of Liver Tumor Growth
Source: Adv Sci (Weinh). 2015 Apr 20;2(5):1500008. doi: 10.1002/advs.201500008 (PMC5115368; doi:10.1002/advs.201500008)
Supplement: Supplementary file 1 — Supplementary [file ADVS-2-0o-s001.pdf]

Copyright WILEY-VCH Verlag GmbH & Co. KGaA, 69469 Weinheim, Germany, 2015.

ADVANCED SCIENCE
Open Access

Supporting Information

for *Adv. Sci.*, DOI: 10.1002/advs. 201500008

Far-Red/Near-Infrared Conjugated Polymer Nanoparticles for Long-Term In Situ Monitoring of Liver Tumor Growth

Jie Liu, Kai Li,* and Bin Liu*

Copyright WILEY-VCH Verlag GmbH & Co. KGaA, 69469 Weinheim, Germany, 2013.

Supporting Information

Far-Red/Near-Infrared Conjugated Polymer Nanoparticles for Long-Term In-Situ Monitoring of Liver Tumor Growth

Jie Liu, Kai Li, and Bin Liu**

***Calculation of PIDT-DBT-Tat NP concentration*:**

The PIDT-DBT-Tat NPs stock solution (5 mL) was freeze-dried to yield 1.2 mg of powders. As the PIDT-DBT-Tat NPs have good colloidal stability in water without precipitation, the density of the NP suspension could be estimated as ~1 g/cm^3. The average size of PIDT-DBT-Tat NPs determined from TEM is ~49 nm. Thus, the concentration of the PIDT-DBT-Tat NPs in stock can be calculated from the following equation:

$$\text{Total number of PIDT-DBT-Tat NPs in 5 mL of suspension}$$

$$= \frac{\text{Total Volume of PIDT-DBT-Tat NPs}}{\text{Average Volume of Each NP}} = \frac{\dfrac{1.2\times10^{-3}\,\text{g}}{1\text{g/mL}}}{\dfrac{4}{3}\pi\times(24.5\times10^{-7})^3\,\text{mL}} = 1.9\times10^{13}$$

Finally, the concentration of PIDT-DBT-Tat NPs in stock solution was calculated as following: $$[\text{PIDT-DBT-Tat NP}] = \frac{\dfrac{2.3\times10^{13}}{6.02\times10^{23}\,\text{mol}^{-1}}}{2\times10^{-3}\,\text{L}} = 16\,\text{nM}$$

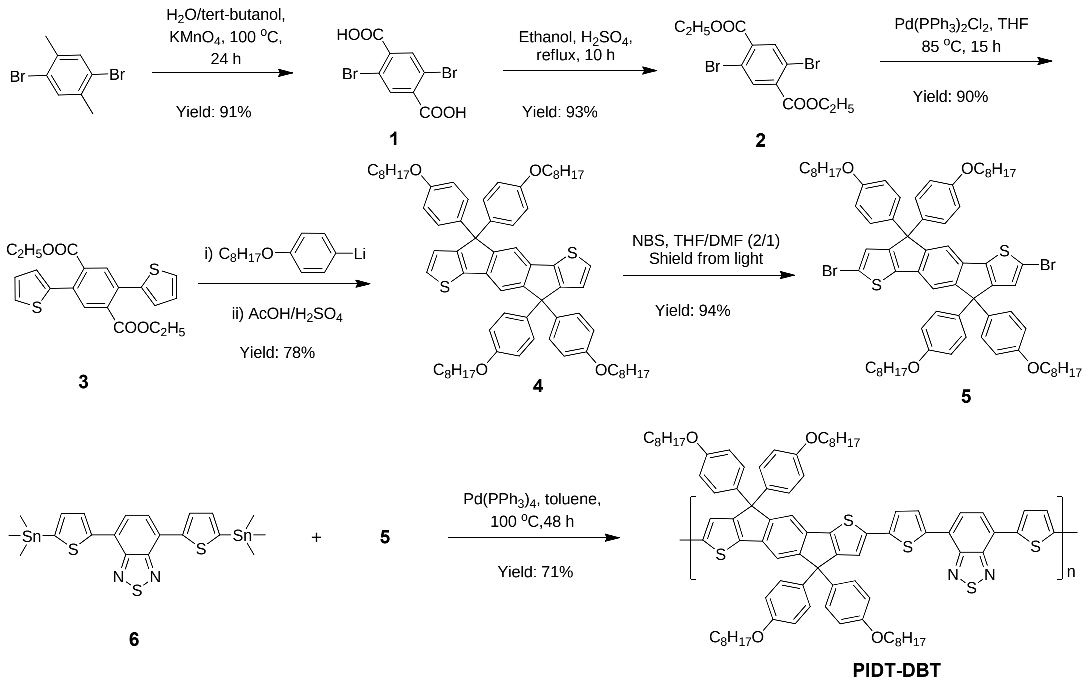

Scheme S1. Synthetic route towards PIDT-DBT.

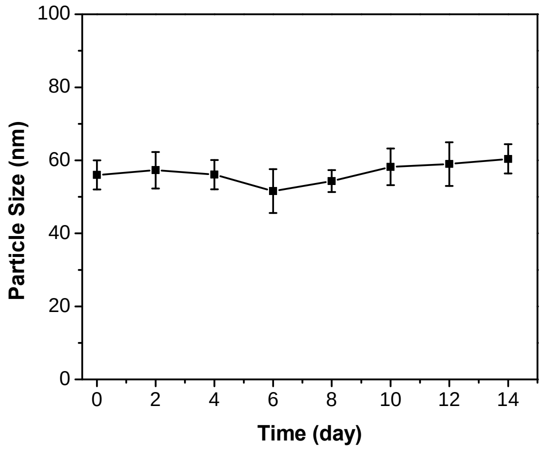

Figure S1. Average hydrodynamic diameter change of PIDT-DBT-Tat NPs when incubating in phosphate-buffered saline at 37 °C for 14 days.

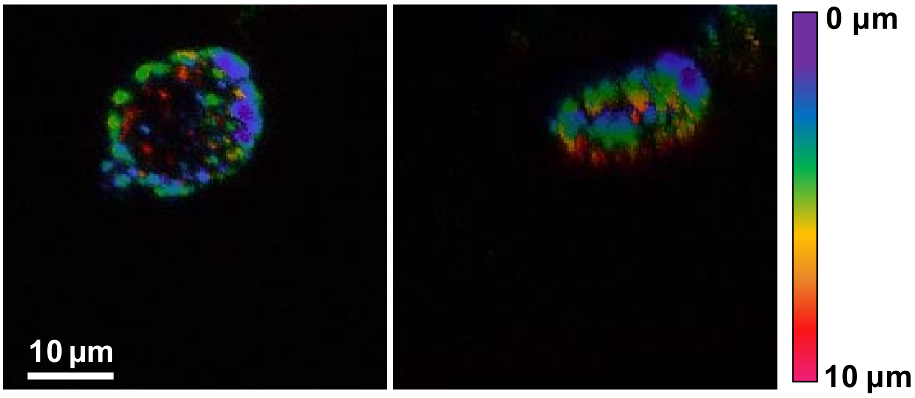

Figure S2. Color-coded projections of z-stacks of confocal images of HepG2 cells upon incubation with PIDT-DBT-Tat NPs overnight. (λ_{ex} = 590 nm, 600-800 nm bandpass filter).

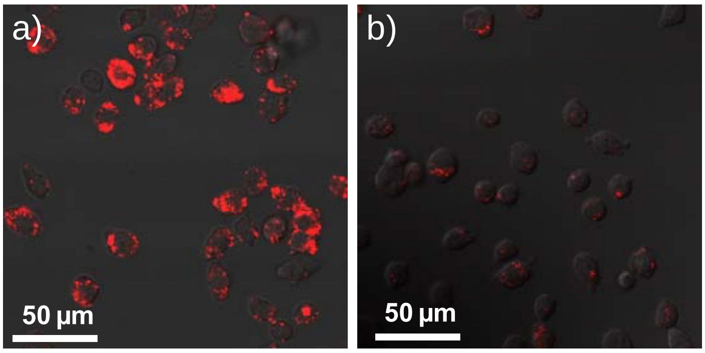

Figure S3. CLSM fluorescence/transmission overlay images of HepG2 cancer cells after incubation with 2 nM (a) PIDT-DBT-Tat NPs and (b) PIDT-DBT NPs at 37 °C for 24 h. (λ_{ex} = 561 nm, 710/50 nm bandpass filter).

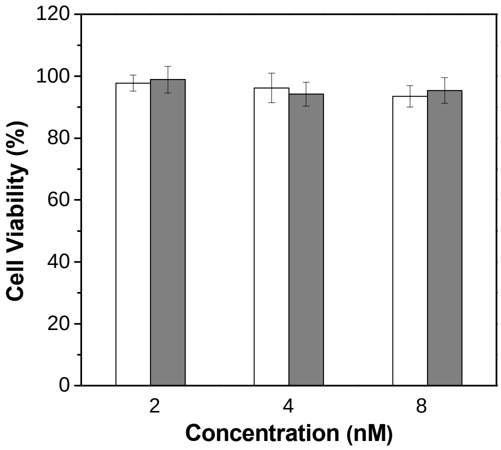

Figure S4. Metabolic viability of HepG2 cells (blank) and NIH/3T3 cells (gray) after incubation with 2, 4 and 8 nM PIDT-DBT-Tat NPs for 48 h.

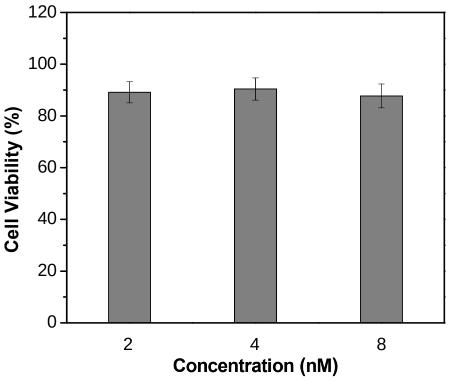

Figure S5. Metabolic viability of NIH/3T3 cells after incubation with 2, 4 and 8 nM PIDT-DBT-Tat NPs for 72 h.

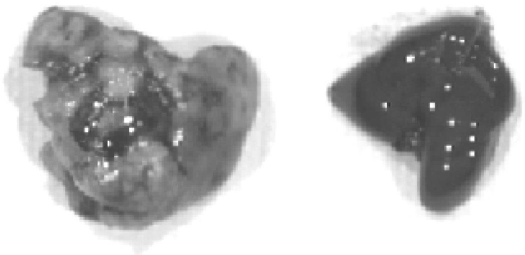

Figure S6. Bright-field image of the liver collected from mouse transplanted with 4×10^6 of PIDT-DBT-Tat NP-labeled HepG2 cells (left) and mouse without transplantation (right) after 42-day injection.

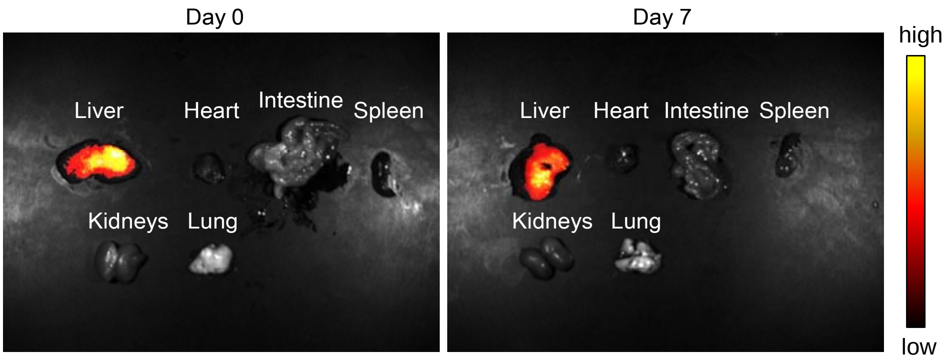

Figure 7. Representative *ex vivo* fluorescence images of the organ tissues collected from mouse transplanted with 4×10^6 of PIDT-DBT-Tat NP-labeled HepG2 cells after 0 and 7 days post injection.
